# Adjuvanted Immunotherapy Approaches for Peanut Allergy

**DOI:** 10.3389/fimmu.2018.02156

**Published:** 2018-09-25

**Authors:** Brandi T. Johnson-Weaver, Herman F. Staats, A. Wesley Burks, Michael D. Kulis

**Affiliations:** ^1^Department of Pathology, Duke University School of Medicine, Durham, NC, United States; ^2^Department of Immunology, Duke University School of Medicine, Durham, NC, United States; ^3^Duke Human Vaccine Institute, Duke University School of Medicine, Durham, NC, United States; ^4^Division of Allergy, Immunology, and Rheumatology, Department of Pediatrics, University of North Carolina at Chapel Hill, Chapel Hill, NC, United States; ^5^University of North Carolina Food Allergy Initiative, Chapel Hill, NC, United States

**Keywords:** food allergy, peanut allergy, immunotherapy, adjuvants, vaccine

## Abstract

Food allergies are a growing public health concern with an estimated 8% of US children affected. Peanut allergies are also on the rise and often do not spontaneously resolve, leaving individuals at-risk for potentially life-threatening anaphylaxis throughout their lifetime. Currently, two forms of peanut immunotherapy, oral immunotherapy (OIT) and epicutaneous immunotherapy (EPIT), are in Phase III clinical trials and have shown promise to induce desensitization in many subjects. However, there are several limitations with OIT and EPIT, such as allergic side effects, daily dosing requirements, and the infrequent outcome of long-term tolerance. Next-generation therapies for peanut allergy should aim to overcome these limitations, which may be achievable with adjuvanted immunotherapy. An adjuvant can be defined as anything that enhances, accelerates, or modifies an immune response to a particular antigen. Adjuvants may allow for lower doses of antigen to be given leading to decreased side effects; may only need to be administered every few weeks or months rather than daily exposures; and may induce a long-lasting protective effect. In this review article, we highlight examples of adjuvants and formulations that have shown pre-clinical efficacy in treating peanut allergy.

## Introduction

Food allergies are common, now estimated to affect 8% of children and 3–6% of the general population in the US (1, 2). The prevalence of food allergies has increased over the past 20 years, although reasons for this increase remain unclear. Peanut allergy is of particular interest because it is typically a life-long allergy (3); in contrast to milk and egg allergies that often spontaneously resolve in childhood. Additionally, peanuts, along with tree nuts, cause the highest number of fatal and near-fatal reactions to foods (4). The prevalence of peanut allergy in the US is estimated at 1–2%, with one ongoing survey finding an increase from 0.4% in 1997 to 1.4% in 2008 (5). There are now 17 named peanut allergens with Ara h 1, 2, 3, and 6 being the major allergens implicated in patients with peanut-induced anaphylaxis (6). The biochemical nature and protein sequences of these allergens have been determined and the genes cloned for recombinant production making development of therapeutics more readily achievable (7, 8).

There are currently no FDA-approved therapies to treat food allergies, limiting allergic individuals to carry injectable epinephrine to treat reactions from accidental exposures. Many types of IgE-mediated allergies are successfully treated with allergen immunotherapy, which has been practiced for over a century, typically administered as subcutaneous injections of soluble allergen extracts (9). Although rather effective for treating environmental and insect venom allergies, subcutaneous injections with peanut extract were tested over 20 years ago and were determined to be unsafe due to high rates of anaphylaxis following injections leading to abandonment of this approach (10, 11). Over the past 10 years, investigators have altered the route of administering allergen immunotherapy to treat food allergy with the intention of minimizing side effects while continually exposing allergic individuals to the offending allergen. These routes of antigen administration include oral ingestion of the allergen (Oral Immunotherapy; OIT), sublingual administration of a soluble protein extract (Sublingual Immunotherapy; SLIT), and epicutaneous administration of dried allergens to the skin (Epicutaneous Immunotherapy; EPIT). Each of these treatment modalities has advantages and disadvantages and each are extensively reviewed by Feuille and Nowak-Wegrzyn (12).

## The promise of adjuvants in food allergy

Undoubtedly, while this is an exciting time in food allergy research as there are two forms of immunotherapy, OIT and EPIT, in Phase III clinical trials, it is equally important to realize the limitations of these approaches. The major limitations are allergic side effects, daily dosing requirements, and ultimately only inducing desensitization and not long-term tolerance. There is room to improve upon these current approaches and one of these ways may be to include immunologic adjuvants. An adjuvant can be defined as anything that enhances, accelerates or modifies an immune response to a particular antigen (13). Adjuvants may allow for lower doses of antigen to be given leading to decreased side effects; may only need to be administered every few weeks or months rather than daily exposures; and may induce a long-lasting protective effect. Aluminum salts (alum) are the most widely used adjuvants for vaccines and have been historically used in subcutaneous immunotherapy formulations to treat respiratory and venom allergies (14). However, alum may not be suitable for peanut therapy due to the severe adverse events associated with subcutaneous peanut immunotherapy and the requirement of prolonged alum exposure to decrease pro-allergic responses (14). Alum may initially exacerbate immunotherapy adverse effects by enhancing allergen-specific IgE antibodies until allergen-specific IgG antibodies that block IgE responses are increased with repeated alum exposures (15). Although alum adjuvants may not be ideal for peanut immunotherapy, there are other encouraging examples of adjuvant utility in peanut immunotherapy. One small OIT trial demonstrated an advantage of a probiotic administered simultaneously in subjects receiving peanut OIT (16). It appears that this method induces a longer-lived state of desensitization, although much larger studies are needed to confirm these initial findings. However, the use of adjuvanted peanut immunotherapy is in its infancy possibly due to the low number of adjuvants approved for use in humans. Prophylactic and therapeutic peanut allergy animal models are useful tools that can be used to identify potential adjuvants that modify host peanut-specific immune responses toward desensitization and/or sustained unresponsiveness to improve the current limitations of peanut allergy immunotherapy.

## Pre-clinical studies of adjuvanted peanut immunotherapy

**Figure 1:**
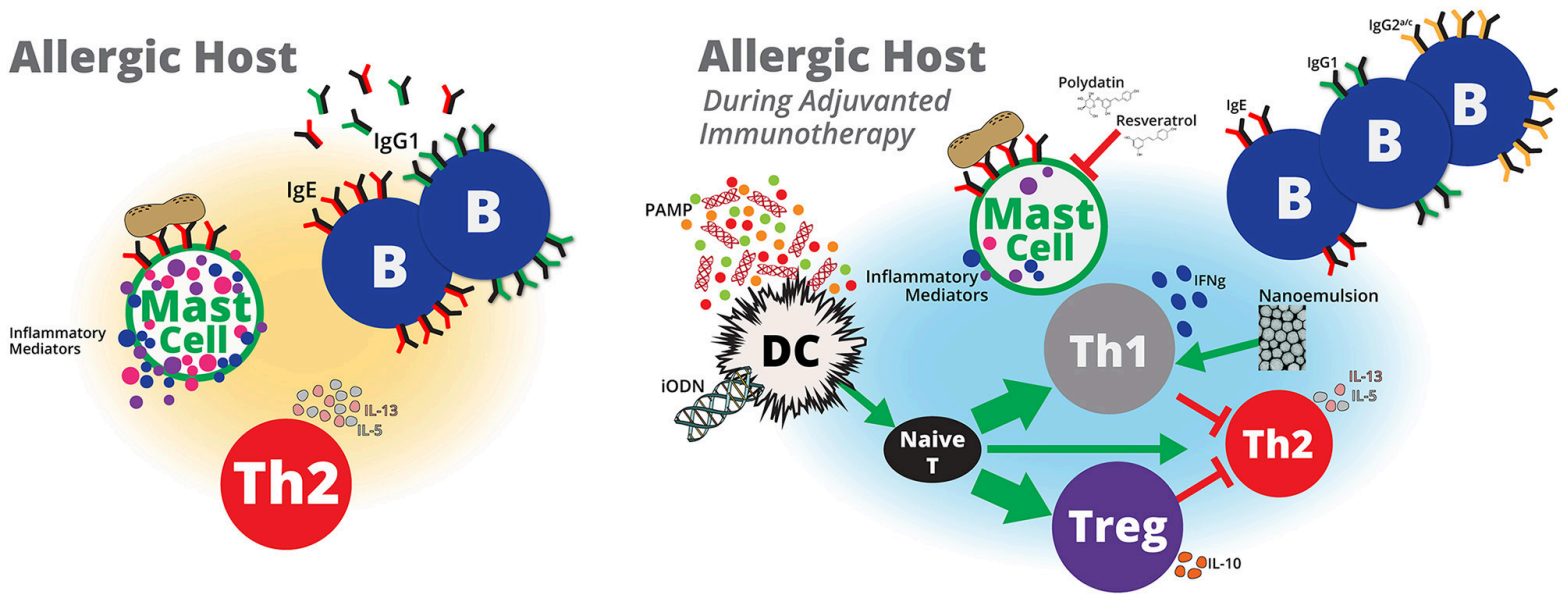
Adjuvanted immunotherapy formulations are potential next-generation therapies that may modulate host immunity to treat peanut allergy. Pathogen-associated molecular patterns (PAMP) derived from pathogens, such as *E. coli* or *L. monocytogenes*, or probiotics, such as *Lactobacillus* and *Bifidobacterium*, or microbial components, such as CpG ODN may activate dendritic cells to induce naïve T cells to differentiate into more Th1 and/or Treg cells and less toward Th2 cells. Inhibitory ODNs that lack CpG and nanoemulsions may also lead to enhanced Treg and Th1 responses, respectively. Increased Th1 and/or Treg cells may suppress or balance peanut-specific Th2 responses to induce protective immunity against peanut. A reduction in peanut-specific Th2 immunity will also reduce the number of peanut-specific IgG1 and IgE antibodies. IgE bound to mast cells cross-linked by peanut antigens induces mast cell activation, which contributes to allergy symptoms. Adjuvants that reduce peanut-specific IgE or stabilize mast cells may prevent therapy-related adverse events and allow for higher doses of peanut to generate protective peanut-specific immune responses. Although immunotherapy with peanut alone appears to be a promising therapy to treat peanut hypersensitivity, safety and the lack of sustained unresponsiveness are potential limitations that may be addressed with adjuvants. Inclusion of vaccine adjuvants to peanut immunotherapy formulations offer potential benefits to peanut immunotherapy that that include, induction of durable protective immune responses in shorter dosing regimens with minimal adverse events.

Animal models of peanut allergy represent invaluable tools to evaluate novel therapeutics or vaccines that may increase the safety and efficacy of peanut immunotherapy. Several types of vaccine adjuvants, including microbial by-products, nanoparticles and nanoemulsions have been evaluated to treat peanut hypersensitivities in animals (Figure 1). However, additional adjuvants that modify mast cell immunity, enhance Th1 and/or T regulatory cell responses, including interferon (IFN)-γ and/or interleukin (IL)-10, respectively or utilize host machinery to down-regulate pro-allergy phenotypes, including Th2-associated IL-4, IL-5, and IL-13, may be beneficial for treating peanut allergy. In the following sections, we will discuss animal studies that describe beneficial effects of adjuvanted immunotherapy to treat peanut hypersensitivity. Additionally, we will discuss adjuvants used in vaccine or non-peanut allergy studies that induce immune responses that may be beneficial for peanut allergy immunotherapy.

### Microbial adjuvants

The magnitude of microbial exposure is thought to modulate allergic disease. Early studies of peanut allergy demonstrated that reduced microbial exposure through antibiotic use or lack of toll-like receptor (TLR) 4 signaling enhanced peanut-induced anaphylactic reactions in mice (17). It is possible that reduced microbial exposure or inability to respond to microbes increases responses associated with allergic disease. For example, mice that lack bacterial colonization (germ-free) have enhanced T helper cell type 2 (Th2) responses and more severe allergic disease than conventional mice (18). While microbial stimulation may be important for proper immune development and allergy regulation, not all bacteria have the same function. A consortium of *Clostridia* from the XIVa, XIVb, and IV clusters isolated from conventional mice but not *Bacteroides uniformis*, enhances Tregs and reduces allergic disease severity (18), which suggests that specific bacteria are important for allergy modulation. Since allergy is mediated by Th2 responses, immune modulators that reduce Th2 responses and increase Treg responses, such as specific commensal bacteria, may be effective immunotherapy adjuvants.

Routine probiotic consumption may be an acceptable method to acquire immune modulating microbes that enhance the efficacy of peanut allergy immunotherapy. Probiotics are considered “good bacteria” and are frequently used to treat intestinal irregularities, including diarrhea. Lactobacillus and Bifidobacterium species are common bacteria used for probiotics (19) and may improve peanut allergy. Oral delivery of a proprietary cocktail of probiotics containing strains of both Lactobacillus and Bifidobacterium successfully reduced allergic disease symptoms in peanut-hypersensitive mice (20). Reductions in allergy symptoms were accompanied by an increase in IL-10 and transforming growth factor (TGF)-β, which are cytokines associated with Treg cells and a decrease in the Th2 cytokine IL-13 (20). Similarly, gastric exposure to Lactobacillus before and during peanut-sensitization reduces peanut-specific IgE, Th2-associated cytokines and mast cell degranulation in mice (21). Bacteria can also be engineered to express peanut allergens and serve as “peanut-vaccines” that utilize self-components to modulate host immunity. *Lactococcus lactis* that produce the peanut protein, Ara h 2, decrease peanut-specific IL-4 and IL-10 responses and increase IFN-γ in mice immunized prophylactically before sensitization compared to mock-immunized mice (22). Probiotics may exert their allergy protective effects by inducing and sustaining Treg responses through their natural components that activate host toll-like receptors (TLR). The TLR ligands present in probiotic bacteria may activate host cells to secrete immunosuppressive cytokines, including TGF-β, which supports Treg differentiation and binds receptors on dendritic cells (DCs), specifically DC-SIGN to enhance IL-10 producing Tregs (23). Probiotic metabolism may generate metabolites that also activate Tregs through G protein-coupled receptors (23). Tregs have been associated with positive outcomes of peanut immunotherapy (24) and probiotics, such as *Lactobacillus acidophilus, L. casei* and *Bifidobacterium bifidium* increase Treg cell numbers and their suppressive functions (25). Enhanced probiotic use should be carefully monitored since probiotics are live cultures that may also influence host microbiota and potentially lead to off-target effects including, excessive immune stimulation, alternative metabolic activities and potential infections in susceptible populations (26, 27). However, probiotics are often used as nutritional supplements and are generally well tolerated (27); therefore, they may be a safe and noninvasive method to favorably modulate the protective immune responses induced by peanut immunotherapy.

Vaccine vectors generated from common pathogens that infect the gastrointestinal (GI) tract have been engineered to express antigens from different sources, including peanut. Similar to probiotic bacteria, these vectors contain pathogen-associated molecular patterns (PAMPs), such as unmethylated CpG DNA, lipoproteins and lipopolysaccharides that can activate the host immune system (28). Since these pathogens have developed mechanisms to evade host immunity to cause infections, their use as attenuated or inactivated vaccine vectors may be beneficial for treating peanut allergy. Peanut-hypersensitive mice treated with three weekly rectal immunotherapy doses of heat-killed *E. coli* (HKE) expressing Ara h 1, 2 and 3 developed decreased peanut-induced IL-4,−5,−13, and−10, increased TGF-β and IFN-γ and less severe allergic symptoms in compared to sham-treated animals (29). While creating genetically modified bacteria may be time-consuming, a more simple approach to treating allergy may combine inactivated pathogens with a known allergen dose in an immunotherapy formulation. Immunotherapy with heat-killed *Listeria monocytogenes* (HKLM) combined with Ara h 1, 2, and 3 administered subcutaneously three times a week for 4 weeks to peanut-hypersensitive mice reduced peanut-induced hypothermia and allergy symptoms (30). Interestingly, the protective effects of HKLM for peanut allergy have also been observed in a dog model. HKLM combined with peanut required higher doses of peanut to induce an allergic reaction in animals with a known history of peanut-induced atopy (31), suggesting that the presence of the bacteria increases the activation threshold required for peanut to induce an allergic response. Although animal models support the use of inactivated pathogenic bacteria as adjuvants to improve peanut allergy, it is possible that host inflammatory responses to these bacteria will induce adverse events while modifying pro-allergic Th2 responses. Human studies demonstrated severe adverse reactions, such as throat discomfort, severe abdominal pain and anaphylaxis, which required subjects to discontinue to study after rectal administration of heat-killed *E. coli*-producing peanut proteins (32). While both *E. coli* and *Listeria* are potent inducers of Th1-immunity, they may not generate effective T regulatory responses. Peanut immunotherapy may benefit more from Treg-inducing adjuvants than strong Th1-inducing adjuvants that only dilute Th2 responses and potentially induce adverse reactions themselves. Therefore, vectors derived from bacteria that may cause gastroenteritis, such as *E. coli* and *L. monocytogenes*, may enhance immunity that prevent allergic development but may not be safe for treating established allergy due to the potential for inflammatory adverse events.

### Microbial by-product adjuvants

Purified microbial macromolecules, such as DNA, lipopeptides and proteins, may induce similar beneficial immune responses for peanut allergy as whole-cell bacteria without the associated risks. Bacteria and viruses express toll-like receptor ligands that are PAMPS, which activate the host immune system to provide adjuvant activity and may modulate pre-existing immune responses. TLR9 ligands, such as unmethylated CpG oligodeoxynucleotides (ODN), are potent inducers of Th1 (33) and Treg (34) immunity and have been evaluated in mouse models of peanut immunotherapy. Peanut combined with CpG prophylactically and therapeutically reduced peanut-induced anaphylaxis in hypersensitive mice when injected or administered orally (35, 36). Exposure to peanut in the presence of CpG decreased peanut-specific Th2 responses, including IgE, IgG1, IL-5, and IL-13 and increased peanut-specific IFN-γ (36). The Th1-immunity induced by CpG may be beneficial for reducing allergy development but CpG may be more beneficial for peanut immunotherapy clinical studies if they induce Treg responses. CpG activates human dendritic cells to increase costimulatory molecules and naïve T cells stimulated by CpG-activated dendritic cells differentiate into Treg cells that suppress effector T cell responses (34). Although TLR ligands (TLRL) can induce similar Th1-associated immune responses as whole-cell bacteria, such as *E. coli* and *L. monocytogenes*, it is possible that TLRL adjuvants only direct immune responses to the co-administered peanut allergen. TLRL adjuvants that only enhance inflammatory responses to the peanut allergen may induce milder inflammation compared to whole-bacterium adjuvants that induce inflammation to peanut allergens and the bacteria itself. Thereby, TLRL adjuvants may enhance immunotherapy safety since it is possible that increased inflammation will contribute to potential off-target adverse effects. TLR ligands, like CpG, may improve OIT, SLIT, and EPIT by suppressing peanut-specific Th2 responses through an increase in Tregs and inducing *de novo* peanut-specific Th1-associated immune responses that balance the pre-existing peanut-specific Th2 cells, both that may improve the likelihood of sustained unresponsiveness.

Although TLRL appear to be promising adjuvants for peanut immunotherapy, selection of age-appropriate adjuvants should be considered when treating peanut-hypersensitive subjects. Host immunity vary with age in response to TLR stimulation (37, 38). Neonates and infants are often less responsive to CpG than adults (39). While CpG may be a more effective adjuvant in older children and adults, infant peanut immunotherapy may benefit from other PAMP adjuvants such as, R848 combined with trehalose-6,6-dibehenate (TBD), that enhance antigen-specific Th1 responses in young populations (37). If PAMP adjuvants are to be used in peanut immunotherapy, then future research must respect the age of the patient during treatment.

### Vaccine formulations as prospective immunotherapy adjuvants

Proper physical formulation that combines peanut allergens with structures designed to improve mucosal allergen delivery and modify host immunity may enhance peanut immunotherapy safety and efficacy. Nanoparticles are structures smaller than 1,000 nm that can transport vaccine formulations and may exert immunomodulatory activity by reducing antigen degradation, increasing antigen uptake by antigen presenting cells (APC) and prolonging antigen retention (40). Nanoparticle immunomodulatory activity may generate immune responses beneficial for the treatment of peanut allergy. Oral vaccination with peanut-containing nanoparticles in naïve mice induces a balanced peanut-specific Th1:Th2 response with enhanced IL-10 responses (41). Nanoparticle-formulated peanut improved hypersensitive responses in mice that received CpG-adjuvanted peanut OIT in nanoparticles (42). OIT with peanut alone, nanoparticles alone, or CpG-nanoparticles in the absence of peanut did not improve allergic disease in peanut-hypersensitive mice but the combination of peanut, CpG and the nanoparticles reduced peanut-induced anaphylaxis (42). Improved allergic disease accompanied a shift from peanut-specific Th2 responses toward peanut-specific Th1 responses, which supports nanoparticles' immune modulating properties.

Nanoemulsions exert immune modulation and allow for lower vaccine component doses to induce comparable immunity to vaccines in comparison with high doses in saline (43). Thus, immunotherapy may benefit from nanoemulsions that allow for lower doses of peanut to modify peanut-specific immunity and reduce the risk of adverse reactions. Nasal vaccination with peanut formulated in a nanoemulsion reduced the severity of allergy in hypersensitive mice whereas the same peanut dose in saline was ineffective (44). Therefore, the physical formulation of peanut immunotherapy is something that should be considered when designing next-generation immunotherapies.

Nanoformulations, including nanoparticles and nanoemulsions are classified by size but can be synthesized from a vast array of materials, including biodegradable chitosan and poly-D-L-lactide-co-glycolide (PLGA) and non-biodegradable ceramic materials (45). Common nanoformulation components such as, oils and surfactants, may have toxic effects and cause cell death (46). *In vitro* and *in vivo* studies that determine the bio-distribution of nanoformulations will also be important since nanoformulations may accumulate within cellular compartments (40) and alter cellular function (47). The toxicity and safety of materials in nanoformulations must be considered when incorporated in a peanut immunotherapy formulation to reduce the risk of additional adverse events.

Physical and immunomodulation properties of nanoformulations may contribute to the protective activity of immunotherapy. Mucosal routes may be preferred for immunotherapy since mucosal routes are naturally tolerogenic and injection immunotherapy may increase systemic anaphylaxis risks (10). Nanoformulations may protect the immunotherapy vaccine components from degradation or clearance to improve allergic disease after mucosal delivery. Additionally, nanoparticles can be designed to control the time and location of antigens released after administration (48). The frequent clinic visits required during therapy may decrease and improve subject compliance through the use of time-release nanoparticles. Controlling the amount of peanut released during immunotherapy by formulation in nanoparticles may also reduce adverse events by limiting the amount of peanut available to activate mast cells.

### Inhibitory ligand adjuvants preventing peanut sensitization

Host immune machinery could potentially have intrinsic activity that can be manipulated to serve as adjuvants to enhance peanut immunotherapy and inhibit allergic disease. Siglec-engaging tolerance-inducing antigenic liposomes (STALs) reduces peanut hypersensitivity in mice when combined with Ara h 2 (49). STALs are liposomes that bind immunomodulatory receptors, including sialic acid-binding Ig-like lectins found on the surface of many cells, such as monocytes, NK cells and B cells (50). Antigen-containing STALs directed against the Siglec on B cells, CD22, can induce apoptosis in antigen-specific B cells and lead to tolerogenic immune responses (51). Pre-treatment with Ara h 2 STALs that target CD22 significantly reduced Ara h2-specifc IgG1 and IgE and prevented peanut-induced hypothermia. Similarly, fusion molecules that contain Ara h 2 and the Fc region of IgG1 (AHG2) reduced peanut-induced allergic responses in hypersensitive mice (52). Peanut-hypersensitive mice displayed a reduction in hypothermia and clinical symptoms with AHG2 therapy compared to placebo (52). Mast cells contain Fc receptors that can activate or inhibit degranulation in response to antigen crosslinking immunoglobulins on the cell surface (53). It is possible the AHG2 molecule inhibits mast cell degranulation through binding inhibitory FcγRs on mast cells in the presence of peanut proteins that would normally activate mast cells through the FcεRI (52). Pre-treatment of peanut-allergic subjects with synthetic molecules that activate host inhibitory receptors in effector cells, similar to those described above, before beginning peanut immunotherapy regimens could potentially reduce adverse reactions by decreasing the number of IgE producing B cells and inhibiting mast cell degranulation. Practical application may require a mixture of multiple inhibitory molecules that are specific for the various peanut proteins since allergic subjects often have IgE responses to several peanut proteins. However, these molecules could be combined with current immunotherapy regimens and allow higher allergen doses to redirect host allergic immune responses. Pre-clinical studies are required to evaluate these therapies in models of established peanut allergy to advance safety and efficacy.

## Lessons learned from alternative adjuvants that may benefit peanut immunotherapy

Peanut immunotherapy could be improved by adding immune modulating molecules that reduce allergy effector cell functions. Inhibition of mast cells and basophil activity during immunotherapy may allow for delivery of higher doses of peanut that can more effectively induce regulatory T cell responses without the complication of allergic symptoms. Inclusion of molecules that directly modulate T cell responses in the immunotherapy formulation may act directly on the T cells to induce sustained unresponsiveness at a more rapid rate than current peanut immunotherapy protocols. Below we will discuss findings from pre-clinical studies that have evaluated adjuvants that modulate mast cells, basophils and T cells in other allergic disease models. The information gained from these studies may be applicable to improve immunotherapy for peanut allergy.

Mast cells are allergy effector cells that directly cause symptoms subsequent to allergen cross-linking IgE on the cell surface (54), therefore mast cell inhibition may be a powerful tool to suppress allergic responses. Resveratrol is a chemical found within plants that has an anti-inflammatory impact on mast cells (55) and attenuates antigen-IgE mediated mast cell activation (56). Mouse diets supplemented with resveratrol reduced allergic sensitization in a mouse model of ovalbumin (OVA) allergy (57). A reduction in OVA-induced hypothermia and OVA-specific IgE, IL-13 and IFN-γ was observed in mice on a resveratrol-supplemented diet. Polydatin is a glucoside of resveratrol and it stabilizes mast cells and decreases the severity of mast-cell dependent passive cutaneous anaphylaxis in mice (58). Cromolyn is another mast cell stabilizer that suppresses allergic airway inflammation in a mouse model of house dust mite (HDM) inflammation (59). Systemic exposure of cromolyn prior to HDM exposure reduced total inflammatory cell numbers and IL-5 in the serum and BAL of sensitized mice (59). Cromolyn is commonly used in asthmatics and provides relief from allergic rhinitis symptoms (60). Importantly, resveratrol and polydatin are components of dietary supplements and are routinely used in humans, which may support the safety of these molecules to be included in peanut immunotherapy formulations.

Rapamycin is another chemical that is used as an immunosuppressive agent clinically and inhibits mast cell cytokine production in response to FcεRI activation (61). OVA-induced mast cell activation and allergic disease severity, including diarrhea score, clinical symptom score and hypothermia, is reduced with oral administration of rapamycin (62). A dose-dependent exposure to rapamycin prevented mast cell growth in the presence of IL-9, decreased serum antibody and splenic cytokine responses (62), suggesting a global immunosuppressive effect. Thus, inclusion of mast cell stabilizers in a peanut immunotherapy formulation may enhance the safety in peanut immunotherapy clinical trials by suppressing mast cell activity that contributes to adverse reactions, however, precaution using global immune-suppressive therapeutics should be considered to reduce the risk of infections.

Adjuvants that enhance Treg responses may contribute to enhance the safety and efficacy of therapies to treat peanut allergy. Inhibitory oligodeoxynucleotides (iODN), which do not contain CpG sequences, activate plasmacytoid dendritic cells to induce Tregs from naïve T cells (63). Since both Th1 and Th2-associated cytokine responses are reduced after treatment with iODNs (64), additional studies are required to evaluate the specificity of suppressive responses induced by iODNs. While global immune suppression may increase the risk of infections, allergen-specific suppression may improve allergic disease. A mouse model of atopic dermatitis described a reduction in clinical skin score, ear thickness and total IgE after oral therapy with encapsulated iODN (65). Although CpG ODN can enhance Tregs, they are also potent inducers of Th1 immunity. Therefore, replacing CpG ODNs with iODNs may further contribute to allergen immunotherapy protective immune responses without the associated Th1-responses that may predispose to other pathologies, including autoimmunity.

The combination of mast cell stabilizers and molecules that induce Tregs may have an additive effect to improve immunotherapy. While it is unclear how mast cell stabilizers modulate allergen-specific immunity, it is possible that the inhibition of mast cell activation reduces the amount of Th2 cytokines available to contribute to disease since IL-4,−5, and−13 are secreted by mast cells (66). It would also be interesting to evaluate the influence of mast cell stabilizers on Treg cells since resveratrol-exposed mice demonstrated suppressed Th1 and Th2 responses (57). The adjuvant combination of mast cell stabilizers and iODNs that enhance Tregs should be used with careful consideration not to create an immune-suppressive environment but if properly formulated may improve current immunotherapy. The potential benefit of combination therapies including, reduced mast cell degranulation and induction of potent allergen-specific Tregs, may decrease adverse events and lead to sustained unresponsiveness to increase efficacy.

## Conclusion

Peanut OIT, SLIT, and EPIT are showing promise as the first therapies to induce protective immune responses in allergic individuals. While some level of desensitization is often achieved through these forms of immunotherapy, the need for sustained unresponsivess, a reduction in adverse events, and more favorable immunotherapy regimens require the development of additional immunotherapy formulations. Adjuvanted allergen immunotherapy approaches may address the current limitations of OIT, SLIT, and EPIT. Further pre-clinical studies are required to investigate the safety and efficacy of various adjuvanted-peanut immunotherapy prior to clinical testing in peanut allergic subjects.

## Author contributions

All authors listed have made a substantial, direct and intellectual contribution to the work, and approved it for publication.

### Conflict of interest statement

The authors declare that the research was conducted in the absence of any commercial or financial relationships that could be construed as a potential conflict of interest. The reviewer SS and handling editor declared their shared affiliation at time of review.
